# Reply to Comments: Using the Cardio-Ankle Vascular Index (CAVI) or the Mathematical Correction Form (CAVI_0_) in Clinical Practice

**DOI:** 10.3390/ijms21072647

**Published:** 2020-04-10

**Authors:** Bart Spronck, Alexander Jurko, Michal Mestanik, Alberto P. Avolio, Ingrid Tonhajzerova

**Affiliations:** 1 Department of Biomedical Engineering, CARIM School for Cardiovascular Diseases, Maastricht University, 6229ER Maastricht, The Netherlands; b.spronck@maastrichtuniversity.nl; 2 Department of Biomedical Engineering, School of Engineering & Applied Science, Yale University, New Haven, CT 06511, USA; 3 Pediatric Cardiology Martin, Jessenius Faculty of Medicine in Martin, Comenius University in Bratislava, 03601 Martin, Slovakia; ltvsro@gmail.com; 4 Department of Physiology, Jessenius Faculty of Medicine in Martin, Comenius University in Bratislava, 03601 Martin, Slovakia; mestanik@jfmed.uniba.sk; 5 Biomedical Center Martin, Jessenius Faculty of Medicine in Martin, Comenius University in Bratislava, 03601 Martin, Slovakia; 6 Department of Biomedical Sciences, Faculty of Medicine, Health and Human Sciences, Macquarie University, Sydney, NSW 2019, Australia; alberto.avolio@mq.edu.au

We read with great interest Alizargar et al.’s comment on our recent review on biomarkers in cervical cancer and human papillomavirus infection [1,2]. In their comment, they raise important questions and concerns about using cardio-ankle vascular index (CAVI) and CAVI_0_ [3,4]. We would like to take this opportunity to elaborate our considerations in deriving CAVI_0_, and to point out some potential misconceptions on the corrections that CAVI and CAVI_0_ do and do *not* perform.

Due to the nonlinear elastic behavior of the artery wall, a higher blood pressure results in a higher pulse wave velocity (PWV) at the time of measurement [5,6,7]. It is this inherent blood pressure dependency that both CAVI and CAVI_0_ aim to correct for. Notably, different individuals with different blood pressures may show a different PWV due to (1) pressure dependency of PWV, and/or (2) intrinsic (“actual”) differences in arterial stiffness [8]. CAVI and CAVI_0_ only correct—and should only correct—for the first effect. Differences in intrinsic arterial stiffness between individuals, e.g., due to hypertensive remodeling, age, obesity, etc., *should* be reflected in CAVI/CAVI_0_, thus allowing for the use of CAVI and CAVI_0_ to study the effect of such phenomena on arterial stiffness. After all, if a stiffness index did not vary with any of those phenomena, there would be no use in measuring it, and it would not predict any outcome.

In a recent study, Shirai et al. compared CAVI and CAVI_0_ in a large population of normotensive and hypertensive individuals [9]. While the use of large populations should be greatly applauded, all analyses presented in their study are cross-sectional, that is, in each individual, CAVI/CAVI_0_ was measured only once. Therefore, relations with blood pressure observed in such a study will reflect both intrinsic (nonlinear elasticity; within-subject) blood pressure dependency and between-subject differences in intrinsic arterial stiffness. As elaborated in the previous paragraph, CAVI and CAVI_0_ are only meant to correct the former and *not* the latter effect. It is clear, therefore, that cross-sectional studies, while very useful to study population patterns and to derive reference values, cannot be used to assess the performance of CAVI and CAVI_0_ in correcting for the intrinsic blood pressure dependency of PWV [8]. Such studies simply contain both effects mixed together, without a way to disentangle them.

Alizargar et al. point out that Shirai et al. observed a negative, “inexplicable” correlation between CAVI_0_ and diastolic blood pressure (DBP) in healthy individuals, which Shirai et al. then used as a reason to label CAVI_0_ as “inappropriate” [1,9]. Again, this correlation is cross-sectional, and indicates (statistically) that subjects with a higher CAVI_0_ on average have a lower DBP. This could be explained as follows. Subjects with (intrinsically) stiffer arteries typically have a larger pulse pressure than subjects with less stiff arteries. As these subjects were classified as healthy (i.e., not hypertensive), their systolic blood pressure was probably approximately normotensive. The DBP of the individuals with stiffer arteries, however, could well have been lower than that of those with less stiff arteries, which potentially explains the negative cross-sectional correlation between CAVI_0_ and DBP as observed by Shirai et al.

Alizargar et al. also mention that in our initial study, we did not take into account physiological properties of individuals, such as body mass index (BMI) [1,4]. Again, BMI is a cross-sectional property that varies between individuals. CAVI/CAVI_0_ were never aimed to be BMI-independent. In our initial study, we simulated subjects with differing values of intrinsic stiffness. Such differences could be interpreted as being due to BMI, age, calcification, arteriosclerosis, etc. However, the aim of our study was not to investigate the effects of these particular phenomena; it was merely to illustrate how, in a typical study cohort with (patho)physiological differences in intrinsic stiffness among subjects, blood pressure fluctuations could (theoretically) influence CAVI and CAVI_0_ measurements.

Calculation of CAVI involves the use of two scale parameters (*a* and *b*) that were recently disclosed to the public [10]. This disclosure allows for an accurate, exact (instead of estimated [11]) conversion from CAVI to CAVI_0_ [12]. Different combinations of *a*/*b* parameters are used for different CAVI ranges [10], the effects of which were criticized by Ato et al. [13]. Notably, CAVI_0_ does not use such scale parameters. Considering these criticisms, as well as the CAVI-CAVI_0_ discussion, Alizargar et al. suggest that normal PWV and stiffness index β may be more reliable indices than the (less established) CAVI and CAVI_0_ [1]. We agree that PWV indeed is more established, and has the advantage that it is “simpler”, or “closer to the measurement”, but with the trade-off of being inherently blood pressure dependent. Stiffness index β (termed heart-ankle β or haβ for the heart-to-ankle trajectory), CAVI, and CAVI_0_ are all much less blood pressure dependent than PWV [4,10,14], and hence, have the potential of being more “intrinsic” metrics of arterial stiffness. Therefore, potentially, the values of such indices may be more directly interpretable as “arterial stiffness” than PWV values.

To conclude, we would like to re-emphasize that we consider the development and practical application of CAVI, as extensively described by Dr. Shirai and colleagues [3,10], to be innovative and based on sound and robust physiological principles. In our paper and discussion with Dr. Shirai, we aimed to provide additional clarification and suggested improvements by using CAVI_0_ [4,15,16,17,18]. We consider it also important to point out that the difference between statistical corrections based on cross-sectional and longitudinal/repeated measures studies can confound the interpretation of the difference between pressure dependency and intrinsic properties of arterial stiffness.

## Abbreviations

BMI	body mass index
CAVI	cardio-ankle vascular index
CAVI_0_	cardio-ankle vascular index 0
DBP	diastolic blood pressure
haβ	heart-to-ankle stiffness index β
PWV	pulse wave velocity
